# Evaluation of different imaging modalities for axillary lymph node staging in breast cancer patients to provide a personalized and optimized therapy algorithm

**DOI:** 10.1007/s00432-022-04221-9

**Published:** 2022-08-10

**Authors:** Joachim Diessner, Laura Anders, Saskia Herbert, Matthias Kiesel, Thorsten Bley, Tanja Schlaiss, Stephanie Sauer, Achim Wöckel, Catharina Bartmann

**Affiliations:** 1 Department of Obstetrics and Gynaecology, University Hospital, Josef-Schneider-Str. 4, 97080 Würzburg, Germany; 2 Department of Diagnostic and Interventional Radiology, University Hospital, Josef-Schneider-Straße 2, 97080 Würzburg, Germany

**Keywords:** Breast cancer imaging, Positive nodal status, Cross-sectional imaging, Conventional imaging, Post-neoadjuvant therapies, Neoadjuvant therapies

## Abstract

**Purpose:**

The reliable detection of tumor-infiltrated axillary lymph nodes for breast cancer [BC] patients plays a decisive role in further therapy. We aimed to find out whether cross-sectional imaging techniques could improve sensitivity for pretherapeutic axillary staging in nodal-positive BC patients compared to conventional imaging such as mammography and sonography.

**Methods:**

Data for breast cancer patients with tumor-infiltrated axillary lymph nodes having received surgery between 2014 and 2020 were included in this study.

All examinations (sonography, mammography, computed tomography [CT] and magnetic resonance imaging [MRI]) were interpreted by board-certified specialists in radiology. The sensitivity of different imaging modalities was calculated, and binary logistic regression analyses were performed to detect variables influencing the detection of positive lymph nodes.

**Results:**

All included 382 breast cancer patients had received conventional imaging, while 52.61% of the patients had received cross-sectional imaging.

The sensitivity of the combination of all imaging modalities was 68.89%. The combination of MRI and CT showed 63.83% and the combination of sonography and mammography showed 36.11% sensitivity.

**Conclusion:**

We could demonstrate that cross-sectional imaging can improve the sensitivity of the detection of tumor-infiltrated axillary lymph nodes in breast cancer patients. Only the safe detection of these lymph nodes at the time of diagnosis enables the evaluation of the response to neoadjuvant therapy, thereby allowing access to prognosis and improving new post-neoadjuvant therapies.

## Introduction

The axillary lymph node status is considered to be one of the most important prognostic factors regarding the long-term survival of breast cancer (BC) patients (Carter et al. 1989; de Boer et al. 2010). Moreover, the knowledge whether lymph nodes are tumor-infiltrated or not is essential, since nodal involvement has decisive therapeutic consequences such as axillary lymph node dissection (ALND), axillary radiotherapy and neoadjuvant or adjuvant systemic chemotherapy (Rao et al. 2013).

To provide individualized therapeutic options and optimized therapy algorithms for BC patients, it is crucial to perform an exact pretherapeutic evaluation of the nodal status of the axilla. Ultrasound is considered to be the imaging modality of choice for evaluating the axillary lymph node status in BC patients and is usually combined with mammography in the conventional imaging setting (Choi H. Y. et al. 2017). The sensitivity of mammography for detection of nodal involvement is stated below 25.00% (Valente et al. 2012). The sensitivity of ultrasound for axillary staging revealing nodal metastases ranges between 15.00 and 85.00% depending on the literature (Alvarez et al. 2006, Choi H. Y. et al. 2017). CT is not used by default for axillary staging, but can be valuable for detecting infiltrated internal mammary and supraclavicular lymph nodes (Lee et al. 2014). Furthermore, CT imaging is able to prove nodal involvement on basis of morphological features such as irregular cortical thickening or the absence of internal fat density (Kutomi et al. 2014; Uematsu et al. 2001). The diagnostic performance of the MRI for detecting axillary metastases seems promising with a sensitivity ranging from 35.00% up to over 80.00% in systematic reviews (Al-Hattali et al. 2019; Kuijs et al. 2015; Zhou et al. 2018).

Over the past decades, different management strategies have emerged in axillary lymph node surgery favoring less invasive procedures. To that effect ALND has been replaced by sentinel lymph node biopsy in early stages of BC (de Meric de Bellefon et al. 2018). Especially after the publication of the ACOSOG Z0011 and AMAROS—clinical trials which have shown that even in patients with nodal involvement (1 or 2 positive sentinel nodes)—who meet certain criteria—there seems to be no additional benefit of the ALND regarding clinical outcome (Donker et al. 2014; Giuliano et al. 2011). In patients with clinically nodal-negative (cN0) disease, the excision of the sentinel node is considered to be the standard procedure for the evaluation of the axilla with identification rates above 90.00% and false-negative rates (FNR) < 10.00% even after NAC (Shirzadi et al. 2019). However, in patients with node-positive disease who receive NAC and convert into clinically nodal-negative disease (ycN0), identification rates of infiltrated lymph nodes by SNB were lower (89.00%) and false-negative rates were higher (13.00%). Therefore, the sole procedure of SNB is not recommended for these high-risk patients and new surgical strategies for the staging of the axilla are needed (Boughey et al. 2013; Ditsch et al. 2020). For this analysis, we evaluated lymph nodes as cN + according to the definition of the AGO (Arbeitsgemeinschaft Gynäkologische Onkologie) breast committee (Ditsch et al. 2020).

An improvement of the axillary evaluation in patients with initially nodal-positive disease and NAC was accomplished by implementing the targeted axillary dissection (TAD). Caudle and coworkers showed a FNR of 2.00% when removing the sentinel lymph node as well as the clipped lymph node after NAC (Caudle et al. 2016). If the axillary lymph node is detected prior to the start of neoadjuvant chemotherapy, histologically proven and consecutively clipped, targeted axillary dissection is an option to contribute to the de-escalation of axillary surgery and save patients from axillary dissection which is associated with high morbidity (Veronesi et al. 2003). The guidelines of the AGO breast committee have already implemented the option of performing a TAD in clinically node-positive patients who converted into ycN0 after neoadjuvant systemic therapy (Ditsch et al. 2020). If we do not distinguish safely between cN + and cN0 patients in the pretherapeutic setting, we miss the opportunity to offer individualized multidisciplinary therapies like the TAD to patients with nodal involvement. Moreover, the overlook of a tumor-infiltrated lymph node in the neoadjuvant setting entails not only the risk of denying the use of TAD but also the possible identification of lymph nodes, which are still positive after the neoadjuvant therapy. This in turn could prevent the group of BC patients, who did not achieve complete remission and who are at high risk of negative clinical outcome, from receiving a post-NAC. The positive clinical effect of post-neoadjuvant therapies has been proven for the Her2-positive and the triple-negative BC (Masuda et al. 2017; von Minckwitz et al. 2019). For hormone receptor-positive breast cancer, there are promising results (Johnston et al. 2020).

The aim of this study was to evaluate the sensitivity of different pretherapeutic imaging modalities (sonography, mammography, CT and MRI) in nodal-positive BC patients and to find out if there is a further benefit of using cross-sectional imaging (MRI, CT) for pretherapeutic axillary staging compared to conventional imaging such as mammography and sonography (Choi H. Y. et al. 2017). Since the utilization of neoadjuvant chemotherapy is advancing and due to the fact that the diagnostic validity of the axillary staging is less conclusive after the administration of NAC, we chose a retrospective study design including only nodal-positive patients receiving adjuvant chemotherapy.

## Methods

### Study population and data collection

The study population consisted of BC patients having received surgery for BC (breast-conserving surgery or mastectomy) as well as lymph node surgeries (sentinel lymph node biopsy and/or axillary lymph node dissection) in the gynecological clinic of the Wuerzburg University Hospital between 2014 and 2020.

Inclusion criteria were no neoadjuvant therapy and a complete data record for evaluation. Furthermore, only patients with post-operatively tumor-infiltrated lymph nodes based on SNB or axillary dissection material were included. The evaluation of suspicious lymph nodes pre-operatively by nodal biopsy was only carried out in rare individual cases.

The following data were collected from the electronic or paper medical data records: age, sex, menopausal status, body mass index (BMI), pathology report (histological type of BC, pathological tumor as well as lymph node status using the TNM system, estrogen receptor, progesterone receptor and Her2 receptor, Ki-67 and grading) and the results of the lymph node status as well as possible metastases in the different imaging modalities [sonography, mammography, MRI, CT (thorax and abdomen)]. Surrogate definition was used to determine BC subtypes considering the hormone receptors and the Her2 receptor as well as Ki-67 according to the German guideline for BC (Bakker et al. 2019): luminal A (HR positive, Her2 negative, Ki-67 < 25%), luminal B Her2 negative (HR positive, Her2 negative, Ki-67 ≥ 25%), luminal B Her2 positive (HR positive, Her2 positive), Her2 overexpressing (HR negative, Her2 positive) and triple negative (HR negative, Her2 negative).

### Imaging technique and interpretation

The imaging diagnostic for BC (MRI, CT, mammography and ultrasound) patients is carried out by the Department of Diagnostic and Interventional Radiology of the University Hospital Wuerzburg and interpreted by a board-certified radiologist at the time of BC diagnosis. The radiological reports for each patient and each imaging technique were analyzed with regard to the lymph node status. For those reports that did not evaluate the nodal status clearly, the images were re-evaluated by two board-certified radiologists with at least seven years of experience in senology based on an inter-reader agreement as part of this study.

This radiologist was blinded and did not know the pathological result of the axillary evaluation. If the axillary lymph nodes were assessed as suspicious, in the different imaging techniques, we evaluated the nodal status as cN + . Inconclusive axillary lymph node status in imaging diagnostics was also interpreted as cN + .

#### MRI protocol

Breast MRI was performed on a 3.0 T scanner, with dedicated breast coils and with patients lying in a prone position. All protocols followed International guidelines and recommendations and included a T2-weighted sequence and a T1-weighted series acquired before and after the injection of a gadolinium-based contrast agent.

Ultrasound was performed by a specialist in radiology with experience in senology on a commercially available system (S2000 or S1000, Siemens Healthineers), using a 14 MHz probe. The BC patients routinely receive an ultrasound exam including the evaluation of the breast and the axilla with the lymphatic drainage pathways.

In the sonographic assessment of the axillary lymph nodes, the following characteristics were evaluated: nodal shape, size, border and internal architecture. Moreover, color and power Doppler ultrasound was done in some cases evaluate the vascular pattern of lymph nodes and to clarify dignity (Ahuja et al. 2008).

For mammography, we used two different full-field digital mammography systems (Selenia Dimensions or Selenia 3Dimensions, Hologic) with the option of additional tomosynthesis for further characterization of the primary tumor.

For staging purpose, we used contemporary multidetector CT systems (Somatom Force, Somatom Definition AS, Somatom Edge, all Siemens Healthineers) with standard acquisition and reconstruction protocols, including chest and abdomen.

For image analysis and archiving, data were transferred to dedicated Picture Archiving and Communication System (PACS) software (Merlin, Phoenix-PACS). In general, all BC patients at our center receive conventional imaging as standard procedure. Individuals at higher risk of metastasis formation receive additional chest and abdominal CT imaging for staging purpose. Patients with germline mutations, such as BRCA1 or BRCA2, with invasive lobular carcinoma, with multicentric BC or with a high density of the breast tissue receive additional MR mammography.

## Statistical analysis

The software IBM SPSS Statistics 26 (IBM Deutschland GmbH, 71,137 Ehningen) was used to collect data, create tables and to perform statistical analysis. Data are presented in numbers and percent (%). Spearman rho test was performed to test correlation of the different imaging modalities. Sensitivity was calculated as the proportion (in percent) of correctly identified positive lymph nodes via imaging. Chi-square tests compared possible differences in the number of positive and negative detected lymph nodes of the imaging modalities. Multiple binary logistic regression analyses were performed to detect variables influencing the detection of positive lymph nodes. *p* values lower than 0.05 were considered significant.

## Results

### Basic characteristics of the study population

The data of 382 node positive BC patients with adjuvant treatment between 2014 and 2020 were enrolled in this study. This corresponds to a percentage of 21.90% of all BC patients treated in our department of gynecological oncology during this period. Basic characteristics of the study population are summarized in Table 1.

**Table 1:** Basic characteristics of the study population

		Patients	Imaging modalities					
		All number / (percent)	Sonography number /(percent)	Mammography number (percent)	MRI number (percent)	CT number (percent)	Conventional imaging (sonography and/or mammography) number (percent)	Cross-sectional imaging (MRI and/or CT) number (percent)
Age (in years)	20–39	20 (5.24%)	20 (100%)	20 (100%)	11 (55.00%)	6 (30.00%)	20 (100%)	14 (70.00%)
	40–59	144 (37.70%)	142 (98.61%)	144 (100%)	57 (39.58%)	44 (30.56%)	144 (100%)	76 (52.78%)
	60–79	177 (46.34%)	177 (100%)	177 (100%)	35 (19.77%)	71 (40.11%)	177 (100%)	89 (50.28%)
	> 79	41 (10.73%)	40 (97.56%)	41 (100%)	3 (7.32%)	21 (51.22%)	41 (100%)	22 (53.66%)
Sex	Female	378 (98.95%)	371 (100%)	374 (100%)	105 (100%)	140 (100%)	374 (100%)	198 (100%)
	Male	4 (1.05%)	4 (100%)	4 (100%)	1 (75.00%)	1 (25.00%)	4 (100%)	2 (50.00%)
Menopausal status	Premenopausal	67 (17.72%)	67 (100%)	67 (100%)	33 (49.25%)	22 (32.84%)	67 (100%)	40 (59.70%)
	Perimenopausal	22 (5.82%)	18 (81.82%)	18 (81.82%)	6 (27.27%)	5 (22.73%)	18 (81.82%)	7 (31.82%)
	Postmenopausal	289 (76.46%)	286 (98.96%)	289 (100%)	66 (100%)	113 (39.10%)	289 (100%)	151 (52.25%)
Body mass index (in kg/m^2^)	< 18,5	7 (1.83%)	7 (100%)	7 (100%)	0 (0%)	2 (28.57%)	7 (100%)	2 (28.57%)
	18, 5–24, 9	165 (43.19%)	165 (100%)	165 (100%)	62 (37.58%)	52 (31.52%)	165 (100%)	86 (52.12%)
	25–29, 9	110 (28.79%)	110 (100%)	110 (100%)	29 (26.36%)	40 (36.36%)	110 (100%)	60 (54.55%)
	30–34, 9	63 (16.49%)	62 (98.41%)	63 (100%)	11 (17.46%)	34 (53.97%)	63 (100%)	36 (57.14%)
	35–39, 9	19 (4.97%)	19 (100%)	19 (100%)	2 (10.53%)	6 (31.58%)	19 (100%)	8 (42.11%)
	> 40	15 (3.93%)	13 (86.67%)	15 (100%)	2 (13.33%)	6 (40.00%)	15 (100%)	7 (46.67%)

*CT* computed tomography, *MRI* magnetic resonance imaging

Most BC patients were suffering from invasive carcinoma of no special type (304 patients; 80.85%), while 57 patients (15.16%) had an invasive lobular breast cancer. Other BC entities were found in 15 patients (3.99%). Further details of stage and subtype of BC are shown in Table 2.

**Table 2:** Pathological stage and subtype of breast cancer

		All patients	Imaging modalities	
		Number (percent of all patients)	Conventional imaging (sonography and/or mammography) number (percent)	Cross-sectional imaging (MRI and/or CT) number (percent)
Intrinsic subtype	Luminal A	220 (57.59%)	220 (100%)	114 (51.82%)
	Luminal B, Her2 neg	91 (23.82%)	91 (100%)	51 (56.04%)
	Luminal B, Her2 pos	35 (9.16%)	35 (100%)	22 (62.86%)
	Her2 overexpressing	11 (2.88%)	11 (100%)	4 (36.36%)
	Triple negative	23 (6.02%)	23 (100%)	9 (39.13%)
	Unknown	2 (0.52%)	2 (100%)	1 (39.13%)
Ki-67 (in %)	0–25	237 (62.86%)	237 (100%)	119 (50.21%)
	26–50	92 (24.40%)	92 (100%)	52 (56.52%)
	51–75	31 (8.22%)	31 (100%)	18 (58.06%)
	76–100	17 (4.51%)	17 (100%)	9 (52.94%)
Grading	1	23 (6.05%)	23 (100%)	11 (47.83%)
	2	254 (66.84%)	254 (100%)	136 (53.54%)
	3	103 (27.11)	103 (100%)	54 (52.43%)
Pathological tumor size using the TNM system	1	131 (34.29%)	131 (100%)	55 (41.98%)
	2	169 (44.24%)	169 (100%)	86 (50.89%)
	3	44 (11.52%)	44 (100%)	33 (75.00%)
	4	33 (8.64%)	33 (100%)	24 (72.73%)
	Unknown	5 (1.31%)	5 (100%)	3 (60.00%)
Lymph node status using the TNM* system	1	266 (69.63%)	266 (100%)	127 (47.74%)
	2	69 (18.06%)	69 (100%)	48 (69.57%)
	3	47 (12.30%)	47 (100%)	26 (55.32%)
Metastasis**	No	344 (90.05%)	344 (100%)	175 (50.87%)
	Yes	38 (9.95%)	38 (100%)	26 (68.42%)

*CT* computed tomography, *MRI* magnetic resonance imaging

^*^ TNM classification is based on the pathological report of breast-conserving therapy or mastectomy and SLN or axillary dissection

pT1: 0–2 cm; pT2: 2–5 cm; pT3: > 5 cm; pN1: 1–3 infiltrated lymph nodes pN2: 4–9 infiltrated lymph nodes pN3: > 9 infiltrated lymph nodes

^**^ Metastasis is defined as a distant dissemination of metastasis detected by imaging techniques

## Different imaging modalities

With regard to the imaging, 379 patients (99.21%) received a sonography and 363 (95.03%) a mammography. Altogether, all patients (382 patients) had a conventional imaging (sonography and/or mammography). While MRI was performed in 106 patients (27.75%), a CT was performed in 142 cases (37.17%). During the investigated period 52.61% of the patients had a cross-sectional imaging (MRI and/or CT). There was a trend of a higher ratio of cross-sectional imaging in relation to conventional imaging (sonography and/or mammography) (Table 3).

**Table 3:** Imaging modalities during the investigated period

	2014	2015	2016	2017	2018	2019	2020	All
Conventional imaging (CI; sonography and/or mammography)	66	43	52	58	56	64	43	382
Cross-sectional imaging (CSI: MRI and/or CT)	16	14	19	31	38	52	31	201
Ratio CSI/CI	0.24	0.33	0.37	0.53	0.68	0.81	0.72	0.53

In 2014, the ratio was 0.24, in 2020, however, 0.72—therefore, the utilization of cross-sectional imaging increased during the last five years.

Bivariate correlation tests revealed correlation coefficients between 0.257 and 0.549 of the results of the different imaging modalities among each other with a significant correlation; p-value was between 0.000 and 0.010 and can, therefore, be considered as highly significant (Table 4).

**Table 4:** Results of the Spearman rho test of the different imaging modalities

Spearman rho		Sonography	Mammography	MRI
Sonography	Correlation coefficient	1	0.416	0.347
	*p* (2–sided)		0.000	0.000
	Number	379	360	106
Mammography	correlation coefficient	0.416	1	0.257
	*p* (2–sided)	0.000		0.010
	Number	360	363	99
MRI	Correlation coefficient	0.347	0.257	1
	*p* (2–sided)	0.000	0.010	
	Number	106	99	106
CT	Correlation coefficient	0.549	0.262	0.427
	*p* (2–sided)	0.000	0.002	0.003
	number	140	139	47

## Sensitivity

Comparing the different imaging modalities, sonography had a sensitivity of 34.56% to detect a positive lymph node status. The sensitivity of the mammography was considerably lower (14.60%). A higher sensitivity was analyzed for MRI with 41.51 and 51.40% for CT. Altogether, the sensitivity of the cross-sectional imaging (MRI and/or CT) was higher than the sensitivity of the conventional imaging (sonography and/or mammography) (50.25 versus 37.17%).

By combination of all imaging modalities, the sensitivity was 68.89%. Slightly lower values could be achieved for the combination of sonography, MRI and CT (68.09%) and for the combination of mammography, MRI and CT (65.91%). The sensitivity of the combination of both cross-sectional imaging modalities (CT and MRI) was 63.83%. Table 5 shows the sensitivity of the imaging modalities and their combinations in a descending order. Generally, there were significant differences between positive and negative lymph node detection in the chi-square testing by comparing the different imaging modalities and their combinations. Only in a few cases, the results were not statistically significant, although the sensitivity varied strongly. (mammography vs. all imaging *p* = 0.053; mammography vs. sonography, MRI and CT *p* = 0.053; mammography vs. MRI and CT *p* = 0.201).

**Table 5:** Sensitivity of the different imaging modalities

	Negative nodal status in at least one imaging modality	Positive nodal status in at least one imaging modality	All	Sensitivity in percent (%)
All imaging modalities	14	31	45	68.89
Sonography, MRI and CT	15	32	47	68.09
Mammography, MRI and CT	15	29	44	65.91
MRI and CT	17	30	47	63.83
Sonography, mammography and CT	54	83	137	60.58
Sonography and CT	58	82	140	58.57
Mammography and CT	61	78	139	56.12
Sonography and MRI	50	56	106	52.83
CT	69	73	142	51.40
Sonography, mammography and MRI	49	50	99	50.51
Mammography and MRI	55	45	100	45.00
MRI	62	44	106	41.51
Sonography and mammography	230	130	360	36.11
Sonography	248	131	379	34.56
Mammography	310	53	363	14.60

## Factors influencing lymph node detection

The main factors influencing the detection of positive lymph nodes significantly in at least one of all imaging modalities were the tumor size (odds ratio 1.93 [1.43–2.59], *p* = 0.000), the nodal status (odds ratio (1.66 [1.17–2.36], *p* = 0.005) and the tumor grading (odd ratio 2.52 [1.49–4.30], *p* = 0.001). Table 6 demonstrates the relevant variables of the cross-sectional imaging, the conventional imaging and each imaging modality alone. These were the pathological tumor size and tumor grading as well as the level of pathological positive lymph nodes. Additionally tested variables were age, body mass index, intrinsic subtypes of BC (surrogate definition of the estrogen, progesterone receptor and Her2 receptor as well as the Ki-67 proliferation marker (Bakker et al. 2019)), metastasis and histological type of BC. There was no statistically significant correlation between the detection of tumor-infiltrated lymph nodes and different imaging techniques (data not shown).

**Table 6:** Multiple binary logistic regression analysis of variables influencing the detection of positive lymph nodes. Tested variables were age, body mass index, subtypes of BC, grading, stage of tumor size and nodal status according to the TNM guidelines, metastasis and histological type of BC

Imaging modalities	variables	Odds ratio (95% confidence interval)	*p*
All (anyone of all)	Tumor size	1.93 (1.43–2.59)	0.000
	Nodal status	1.66 (1.17–2.36)	0.005
	Grading	2.52 (1.49–4.30)	0.001
Cross-sectional imaging (MRI and/or CT)	Nodal status	2.03 (1.25–3.30)	0.004
	Grading	3.29 (1.49–7.24)	0.003
Conventional imaging (sonography and/or mammography)	Tumor size	1.60 (1.20–2.14)	0.001
	Nodal status	1.56 (1.10–2.20)	0.011
	Grading	2.98 (1.72–5.16)	0.000
Sonography	Tumor size	1.53 (1.14–2.04)	0.004
	Nodal status	1.62 (1.15–2.29)	0.006
	Grading	3.05 (1.75–5.32)	0.000
Mammography	Tumor size	1.93 (1.33–2.80)	0.001
	Grading	2.72 (1.33–5.55)	0.006
MRI	Nodal status	3.45 (1.58–7.52)	0.002
	Grading	4.22 (1.31–13.65)	0.016
CT	Grading	3.43 (1.33–8.87)	0.011

Table 7 illustrates the increasing sensitivity with increasing tumor size for sonography, mammography and CT. MRI, however, shows almost constant sensitivity values independently of the tumor size with a range of 41.46–52.94%. In contrast, sonography had a sensitivity of 18.88% for pT1 (TNM classification), 38.86% for pT2, 51.11% for pT3 and 55.88% for pT4. Except for tumor stage pT4, there were significant differences between conventional imaging (sonography and/or mammography) and cross-sectional imaging (MRI and/or CT). For tumor stage pT1, there was a clearly higher sensitivity of cross-sectional imaging to detect positive lymph nodes.

**Table 7:** Sensitivity of the different imaging modalities depending on tumor size

Sensitivity in percent (%)	T1	T2	T3	T4
At least one positive lymph node in any imaging	24.48	46.02	70.21	72.22
MRI and/or CT	36.51	52.81	61.76	62.96
Sonography and/or mammography	20.28	40.34	57.45	58.33
Sonography	18.88	38.86	51.11	55.88
Mammography	5.84	14.46	24.44	38.24
MRI	41.46	40.48	52.94	50
CT	30.77	56.06	52	60.87

## Imaging example emphasizing the importance of cross-sectional imaging for the evaluation of axillary lymph nodes of BC patients

**Fig. 1:**
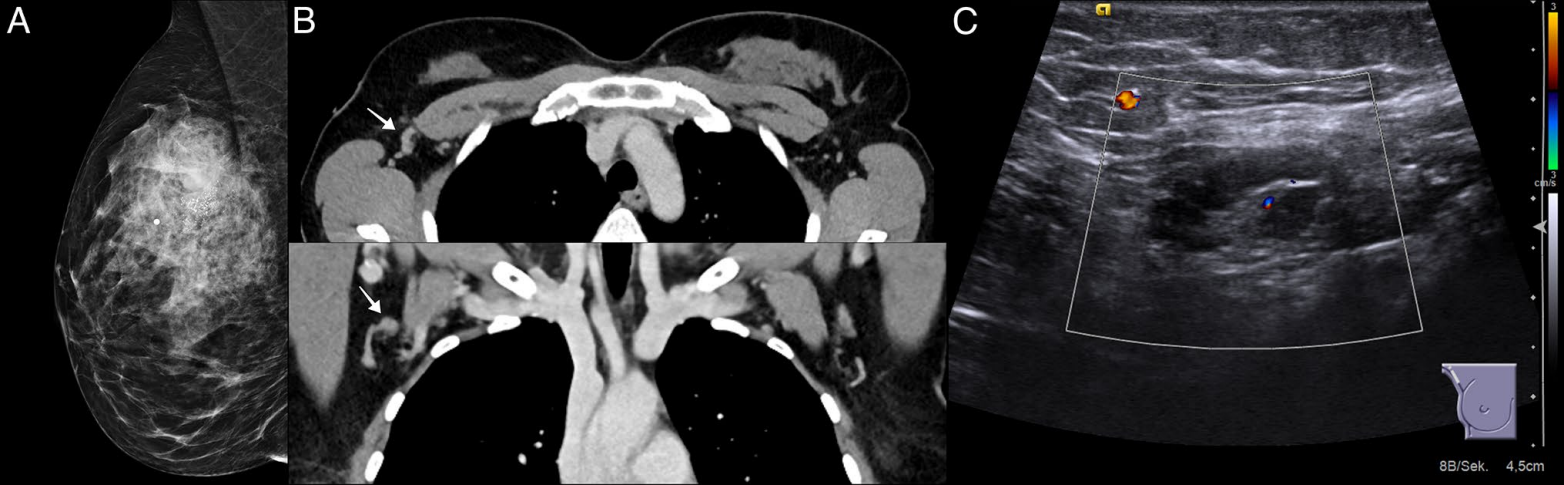
The imaging example illustrates the case of a patient in whom the suspicious lymph node was primarily diagnoses via CT imaging. **A**: Mammography in mediolateral oblique projection depicting invasive breast cancer (no special type, G3) as a mass with architectural distortion and peritumoral ductal carcinoma in situ (DCIS), indicated by accompanying regional distribution of fine linear branching microcalcifications in the right upper outer quadrant. No axillary lymph nodes are included in the field of view. **B**: Axial and coronal reformatting of contrast-enhanced chest CT shows asymmetrical enlargement of one right axillary lymph node with nodular thickening of the cortex (arrows). **C**: Following CT, focused ultrasound of the right axilla confirmed focal nodular thickening, while visualizing an aberrant small vessel within the otherwise homogenously thickened lymph node cortex. Core needle biopsy revealed a nodal metastasis of breast cancer

We present the imaging diagnostics of a 39-year-old patient with a no special type BC with the following TNM classification: pT2 pN1 G3 L1 V1 M 0, Ki-67: 70%, hormone receptor positive, Her2 negative. In this case, the CT at initial staging of thorax and abdomen gave the decisive suspicion of axillary lymph node infiltration. Conventional imaging, however, could not clearly represent the tumor-infiltrated lymph nodes (Fig. 1 A–C).

## Discussion

In the field of senology, there were different trends during the last decade that significantly changed therapy algorithms. On the one hand, there is a trend towards de-escalation of surgical and systemic therapy (de Meric de Bellefon et al. 2018, Mariotto et al. 2020; Tolaney et al. 2015; van der Voort et al. 2021; Veronesi et al. 2002).

On the other hand, there is a trend to escalate systemic therapy for BC patients at high risk. The response to neoadjuvant therapy plays a decisive role in identifying patients with a high risk of relapse. BC patients, who achieve complete pathological response during neoadjuvant therapy, seem to have the best clinical outcome (Huang et al. 2020). Patients with residual tumor after neoadjuvant therapy are, however, at high risk of tumor relapse. This subgroup can benefit from an escalation of cancer therapy by applying post-NAC (Johnston et al. 2020; Masuda et al. 2017; Tutt et al. 2021; von Minckwitz et al. 2019).

The evaluation of the clinical response to NAC in the mammary gland can be achieved by pathological examination of the tumor bed after breast-conserving therapy (von Minckwitz et al. 2019). The assessment of the response of the axillary, respectively, tumor-infiltrated lymph nodes, without performing a complete axillary dissection with its additional side effects is technically challenging.

One of the reasons for this is the fact that the same suspicious lymph nodes have to be analyzed before and after their alteration caused by NAC (Caudle et al. 2016; Simons et al. 2020; Swarnkar et al. 2021). The evolving role of marked lymph node biopsy (MLNB) and targeted axillary dissection (TAD) enables the option of analyzing the effect of NAC. Hence, a statement whether tumor-infiltrated lymph nodes turned negative during therapy can be made (Flores-Funes et al. 2020; Swarnkar et al. 2021). Both mammary and axillary tumor manifestations can develop differently. Because of this, the individual assessment of each of these two tumor localities is crucial (Choi H. J. et al. 2019). The identification of chemotherapy resistant tumor manifestations provides the option of offering post-NAC therapy to selected BC patients (Caparica et al. 2019).

Up to now, the combination of mammography and sonography is the standard of care concerning morphological imaging for BC detection (Okello et al. 2014). Especially, the evaluation of the dignity of lymph nodes is prone to errors. Sonography as the standard technique for lymph node evaluation varies from 15.00 to 85.00% in terms of sensitivity. The specificity, however, is stated to be about 90.00 to 95.00% depending on the literature (Alvarez et al. 2006; Rezvani et al. 2018; Riedel et al. 2021). We, therefore, focused on the sensitivity of different medical imaging procedures: sonography, mammography, MRI and CT and aimed to analyze which technique could improve sensitivity in terms of the detection of tumor-infiltrated lymph nodes. Furthermore, we compared “conventional imaging” consisting of sonography and mammography to cross-sectional imaging consisting of MRI and CT.

Neoadjuvant systemic therapy is becoming increasingly important for treatment of BC worldwide. In addition, tumor-infiltrated lymph nodes often become tumor free after the application of such neoadjuvant therapy. Due to this, we conducted a retrospective analysis and excluded patients with NAC. However, a prospective analysis comparing different imaging techniques for the detection of axillary lymph nodes while excluding patients with neoadjuvant therapies would be highly interesting.

Yet, such a study would be challenging concerning implementation and patient recruitment. For our analysis, we included patients with pathologically confirmed infiltration of axillary lymph nodes.

The analysis of our data showed a sensitivity of 34.56% for sonography and 36.11% for the combination of sonography and mammography for the detection of tumor-infiltrated lymph nodes in the axilla. Our results are within the expected range. It is, however, noticeable that sensitivity for sonography is reported to vary from 15.00 to 85.00% (Alvarez et al. 2006; Rezvani et al. 2018; Riedel et al. 2021; Valente et al. 2012). Elastography may improve sensitivity in this context (Chang et al. 2018; Tsai et al. 2013). In our study, mammography showed a relatively low sensitivity of 14.60% for the detection tumor-infiltrated lymph nodes. The reason for this lies in the insufficient depiction of the axilla by this technique and is comparable to previous results which analyzed a sensitivity of 13.00% (Valente et al. 2012).

For the MRI analysis, we could detect a sensitivity of 41.51 and 63.83% for cross-sectional imaging as a combination of MRI and CT. The diagnostic reliability of MRI for the evaluation of axillary nodal staging in BC reported by previous studies shows inconsistent results. This inconsistency is caused by the monocentric character of many studies, differing MRI examination technology as well as instruments or varying patient inclusion criteria (Al-Hattali et al. 2019; Kuijs et al. 2015; Luo et al. 2013; Zhou et al. 2018). Additionally, in the past, MRI for non-palpable lymph nodes was mostly not focused on the detection of axillary lymph node metastasis in patients with BC. Instead, the reason for an additional MRI was inter alia dense breast tissue or invasive lobular mammary carcinoma (Bakker et al. 2019; Baltzer et al. 2011).

Computed tomography is established for staging exams in wake of BC rather than screening or diagnostic of a mammary tumor itself. The specificity is reported to be about 40.00% (Marino et al. 2020). In our study, we could analyze a sensitivity of 51.40% for the detection of infiltrated lymph nodes for patients who received a CT as staging examination. This sensitivity is slightly lower than reported in the literature (Yuen et al. 2004). However, it has to be considered that the focus of this diagnostic was the detection of distant metastasis (Yuen et al. 2004). In this field, research approaches compared the CT results before and after chemotherapy to evaluate the response in axillary lymph nodes. This investigation could demonstrate promising results (Wang et al. 2021).

The combination of advancing MRI technology and CT staging, both focusing on axillary lymph nodes, could possibly improve sensitivity in the future. As CT examinations of the chest and the abdomen are the standard staging procedure for BC patients with high risk of metastasis formation, this imaging data is already available (Ditsch et al. 2020). During the period of data collection, we could detect an increasing importance of the cross-sectional imaging.

The combination of all four imaging modalities reaches a sensitivity of 68.89%. The combined evaluation of the results as presented in Table 5 depicts clearly that cross-sectional imaging contributes significantly to the increase in sensitivity and underlines its importance.

The regression analysis in Table 6 for evaluating the influence of various clinical parameters on the sensitivity reveals that tumor size, grading and the number of tumor-infiltrated lymph nodes significantly influences the sensitivity of imaging techniques. Large tumor spread, aggressive BC subtypes and heavily tumor-infiltrated axillary lymph nodes lead to a higher probability of being detected correctly. Table 7 illustrates that especially for tumors, smaller than 2 cm (pT1), MRI is the best imaging. Moreover, cross-sectional imaging significantly improves the sensitivity for the detection of infiltrated lymph nodes in relation to conventional imaging for the subgroup of BC patients with pT1 tumors. Whereas CT, mammography and sonography show significantly lower sensitivity values for the detection of tumor-infiltrated lymph nodes for smaller breast tumors than for patients with large tumor spread. Especially, pT1 tumors with high grading are, however, at high risk of developing lymph node metastasis. Especially, for this subgroup of BC patients, carrying out an MRI could be valuable (Zhao et al. 2019).

For other clinical parameters such as age, intrinsic subtype, histological subtype and body mass index [BMI] we could not prove any effect of image modality on the sensitivity of lymph node detection.

One of the main limitations of this analysis is the retrospective data collection. The quality of the study could be improved, if all patients had received all four imaging modalities with focus on axillary infiltration of lymph nodes. Because of the retrospective character of this analysis, we cannot present complete imaging techniques for each patient. Furthermore, the assessment of each imaging technique by the same diagnostician would have reduced interobserver variability. Another limitation of this analysis is the selection bias for imaging techniques. Patients, who received additional MRI or CT imaging are mostly patients with additional risk factors. Therefore, the probability of axillary tumor infiltration is higher for this subgroup of BC patients with cross-sectional imaging. Consequently, on the one hand it seems possible, that in the group of “low-risk” BC patients who did not receive additional cross-sectional imaging some patients with tumor-infiltrated lymph nodes were overlooked by conventional imaging. On the other hand, the effect size for the advantage of cross-sectional imaging might have become lower if all patients had received all four imaging modalities with focus on axillary infiltration of lymph nodes.

Because of the retrospective character of this study, we could not eliminate this statistical problem.

Despite these limitations, we could demonstrate that cross-sectional imaging as MRI and CT in combination with MRI can improve the diagnostic performance for detecting tumor-infiltrated lymph nodes of BC patients. Current literature reports that MRI outperforms mammography and ultrasound for the detection of early breast cancer.

Moreover, it reduces the exposure to radiation as well as the potentially painful compression of the patient’s breast during the imaging process (Mann et al. 2019). Therefore, the increasing use of MRI technology for BC patients could help to detect tumors at an earlier stage and help to identify tumor-infiltrated axillary lymph nodes. The additional focus of CT imaging on axillary lymph nodes could even improve this trend. This could enable the use of technologies such as the marked lymph node biopsy (MLNB) and targeted axillary dissection (TAD). Consequently, patients at high risk, who did not achieve complete remission during NAC, could receive treatment with post-neoadjuvant therapeutic strategies. Currently, some of these critical patients can be deprived of a post-neoadjuvant treatment if tumor-infiltrated lymph nodes in the axilla are not detected at the beginning of cancer therapy.

## Conclusion

In summary, we could demonstrate that cross-sectional imaging with MRI and CT can improve the sensitivity for detecting tumor-infiltrated axillary lymph nodes in BC patients. The increasing importance of NAC and post-NAC therapeutic algorithms in the treatment of BC makes the reliable detection and marking of tumor-infiltrated lymph nodes pivotal. Only the successful detection of a tumor-infiltrated lymph node at the time of diagnosis allows the evaluation of the response to NAC, thus allowing access to prognosis and improving new post-neoadjuvant therapies.

## Data Availability

The data that support the findings of this study are available from Joachim Diessner but restrictions apply to the availability of these data, which were used under license for the current study, and so are not publicly available. Data are however available from the authors upon reasonable request and with permission of Joachim Diessner.

## References

[CR1] Ahuja AT, Ying M, Ho SY, Antonio G, Lee YP, King AD, Wong KT (2008) Ultrasound of malignant cervical lymph nodes. Cancer Imaging 8:48–56 18390388 10.1102/1470-7330.2008.0006 PMC2324368

[CR2] Al-Hattali S, Vinnicombe SJ, Gowdh NM, Evans A, Armstrong S, Adamson D, Purdie CA, Macaskill EJ (2019) Breast MRI and tumour biology predict axillary lymph node response to neoadjuvant chemotherapy for breast cancer. Cancer Imaging 19:91 31878958 10.1186/s40644-019-0279-4 PMC6933687

[CR3] Alvarez S, Anorbe E, Alcorta P, Lopez F, Alonso I, Cortes J (2006) Role of sonography in the diagnosis of axillary lymph node metastases in breast cancer: a systematic review. AJR Am J Roentgenol 186:1342–1348 16632729 10.2214/AJR.05.0936

[CR4] Bakker MF et al (2019) Supplemental MRI screening for women with extremely dense breast tissue. N Engl J Med 381:2091–2102 31774954 10.1056/NEJMoa1903986

[CR5] Baltzer PA, Dietzel M, Burmeister HP, Zoubi R, Gajda M, Camara O, Kaiser WA (2011) Application of MR mammography beyond local staging: is there a potential to accurately assess axillary lymph nodes? Evaluation of an extended protocol in an initial prospective study. AJR Am J Roentgenol 196:W641-647 21512057 10.2214/AJR.10.4889

[CR6] Boughey JC et al (2013) Sentinel lymph node surgery after neoadjuvant chemotherapy in patients with node-positive breast cancer: the ACOSOG Z1071 (Alliance) clinical trial. JAMA 310:1455–1461 24101169 10.1001/jama.2013.278932 PMC4075763

[CR7] Caparica R, Lambertini M, Ponde N, Fumagalli D, de Azambuja E, Piccart M (2019) Post-neoadjuvant treatment and the management of residual disease in breast cancer: state of the art and perspectives. Ther Adv Med Oncol 11:1758835919827714 30833989 10.1177/1758835919827714 PMC6393951

[CR8] Carter CL, Allen C, Henson DE (1989) Relation of tumor size, lymph node status, and survival in 24,740 breast cancer cases. Cancer 63:181–187 2910416 10.1002/1097-0142(19890101)63:1<181::aid-cncr2820630129>3.0.co;2-h

[CR9] Caudle AS et al (2016) Improved axillary evaluation following neoadjuvant therapy for patients with node-positive breast cancer using selective evaluation of clipped nodes: implementation of targeted axillary dissection. J Clin Oncol 34:1072–1078 26811528 10.1200/JCO.2015.64.0094 PMC4933133

[CR10] Chang W, Jia W, Shi J, Yuan C, Zhang Y, Chen M (2018) Role of Elastography in axillary examination of patients with breast cancer. J Ultrasound Med 37:699–707 29344976 10.1002/jum.14538

[CR11] Choi HJ, Ryu JM, Kim I, Nam SJ, Kim SW, Yu J, Lee JE, Lee SK (2019) Prediction of axillary pathologic response with breast pathologic complete response after neoadjuvant chemotherapy. Breast Cancer Res Treat 176:591–596 31065874 10.1007/s10549-019-05214-y

[CR12] Choi HY, Park M, Seo M, Song E, Shin SY, Sohn YM (2017) Preoperative axillary lymph node evaluation in breast cancer: current issues and literature review. Ultrasound Q 33:6–14 28187012 10.1097/RUQ.0000000000000277

[CR13] de Boer M, van Dijck JA, Bult P, Borm GF, Tjan-Heijnen VC (2010) Breast cancer prognosis and occult lymph node metastases, isolated tumor cells, and micrometastases. J Natl Cancer Inst 102:410–425 20190185 10.1093/jnci/djq008

[CR14] de Meric de Bellefon M, Lemanski C, Ducteil A, Fenoglietto P, Azria D, Bourgier C. (2018) Management of the axilla in the era of breast cancer heterogeneity. Front Oncol 8:84 29670853 10.3389/fonc.2018.00084 PMC5893721

[CR15] Ditsch N et al (2020) AGO recommendations for the diagnosis and treatment of patients with locally advanced and metastatic breast cancer: update 2020. Breast Care (basel) 15:294–309 32774225 10.1159/000508736 PMC7383289

[CR16] Donker M et al (2014) Radiotherapy or surgery of the axilla after a positive sentinel node in breast cancer (EORTC 10981–22023 AMAROS): a randomised, multicentre, open-label, phase 3 non-inferiority trial. Lancet Oncol 15:1303–1310 25439688 10.1016/S1470-2045(14)70460-7 PMC4291166

[CR17] Flores-Funes D, Aguilar-Jimenez J, Martinez-Galvez M, Ibanez-Ibanez MJ, Carrasco-Gonzalez L, Gil-Izquierdo JI, Aguayo-Albasini JL (2020) The problem of axillary staging in breast cancer after neoadjuvant chemotherapy. Role of targeted axillary dissection and types of lymph node markers. Cir Esp (engl Ed) 98:510–515 32386728 10.1016/j.ciresp.2020.03.012

[CR18] Giuliano AE, Hunt KK, Ballman KV, Beitsch PD, Whitworth PW, Blumencranz PW, Leitch AM, Saha S, McCall LM, Morrow M (2011) Axillary dissection vs no axillary dissection in women with invasive breast cancer and sentinel node metastasis: a randomized clinical trial. JAMA 305:569–575 21304082 10.1001/jama.2011.90 PMC5389857

[CR19] Huang M et al (2020) Association of pathologic complete response with long-term survival outcomes in triple-negative breast cancer: a meta-analysis. Cancer Res 80:5427–5434 32928917 10.1158/0008-5472.CAN-20-1792

[CR20] Johnston SRD et al (2020) Abemaciclib combined with endocrine therapy for the adjuvant treatment of HR+, HER2-, node-positive, high-risk, early breast cancer (monarchE). J Clin Oncol 38:3987–3998 32954927 10.1200/JCO.20.02514 PMC7768339

[CR21] Kuijs VJ, Moossdorff M, Schipper RJ, Beets-Tan RG, Heuts EM, Keymeulen KB, Smidt ML, Lobbes MB (2015) The role of MRI in axillary lymph node imaging in breast cancer patients: a systematic review. Insights Imaging 6:203–215 25800994 10.1007/s13244-015-0404-2 PMC4376816

[CR22] Kutomi G, Ohmura T, Satomi F, Takamaru T, Shima H, Suzuki Y, Otokozawa S, Zembutsu H, Mori M, Hirata K (2014) Lymph node shape in computed tomography imaging as a predictor for axillary lymph node metastasis in patients with breast cancer. Exp Ther Med 8:681–685 25009640 10.3892/etm.2014.1787 PMC4079443

[CR23] Lee SC, Jain PA, Jethwa SC, Tripathy D, Yamashita MW (2014) Radiologist’s role in breast cancer staging: providing key information for clinicians. Radiographics 34:330–342 24617682 10.1148/rg.342135071

[CR24] Luo N, Su D, Jin G, Liu L, Zhu X, Xie D, Liu Y (2013) Apparent diffusion coefficient ratio between axillary lymph node with primary tumor to detect nodal metastasis in breast cancer patients. J Magn Reson Imaging 38:824–828 23440958 10.1002/jmri.24031

[CR25] Mann RM, Kuhl CK, Moy L (2019) Contrast-enhanced MRI for breast cancer screening. J Magn Reson Imaging 50:377–390 30659696 10.1002/jmri.26654 PMC6767440

[CR26] Marino MA, Avendano D, Zapata P, Riedl CC, Pinker K (2020) Lymph node imaging in patients with primary breast cancer: concurrent diagnostic tools. Oncologist 25:e231–e242 32043792 10.1634/theoncologist.2019-0427 PMC7011661

[CR27] Mariotto A, Jayasekerea J, Petkov V, Schechter CB, Enewold L, Helzlsouer KJ, Feuer EJ, Mandelblatt JS (2020) Expected monetary impact of oncotype DX score-concordant systemic breast cancer therapy based on the TAILORx trial. J Natl Cancer Inst 112:154–160 31165854 10.1093/jnci/djz068 PMC7019096

[CR28] Masuda N et al (2017) Adjuvant capecitabine for breast cancer after preoperative chemotherapy. N Engl J Med 376:2147–2159 28564564 10.1056/NEJMoa1612645

[CR29] Okello J, Kisembo H, Bugeza S, Galukande M (2014) Breast cancer detection using sonography in women with mammographically dense breasts. BMC Med Imaging 14:41 25547239 10.1186/s12880-014-0041-0 PMC4311471

[CR30] Rao R, Euhus D, Mayo HG, Balch C (2013) Axillary node interventions in breast cancer: a systematic review. JAMA 310:1385–1394 24084924 10.1001/jama.2013.277804

[CR31] Rezvani A, Zahergivar A, Iranpour P, Akrami M, Kazemi S (2018) diagnostic accuracy of axillary ultrasonography compared with intra-operative pathological findings in patients with breast cancer. Asian Pac J Cancer Prev 19:3615–3621 30583690 10.31557/APJCP.2018.19.12.3615 PMC6428527

[CR32] Riedel F et al (2021) Diagnostic accuracy of axillary staging by ultrasound in early breast cancer patients. Eur J Radiol 135:109468 33338758 10.1016/j.ejrad.2020.109468

[CR33] Shirzadi A, Mahmoodzadeh H, Qorbani M (2019) Assessment of sentinel lymph node biopsy after neoadjuvant chemotherapy for breast cancer in two subgroups: Initially node negative and node positive converted to node negative - a systemic review and meta-analysis. J Res Med Sci 24:18 30988686 10.4103/jrms.JRMS_127_18 PMC6421883

[CR34] Simons JM, Koppert LB, Luiten EJT, van der Pol CC, Samiei S, de Wilt JHW, Siesling S, Smidt ML (2020) De-escalation of axillary surgery in breast cancer patients treated in the neoadjuvant setting: a Dutch population-based study. Breast Cancer Res Treat 180:725–733 32180074 10.1007/s10549-020-05589-3 PMC7103007

[CR35] Swarnkar PK, Tayeh S, Michell MJ, Mokbel K (2021) The Evolving role of Marked lymph node biopsy (MLNB) and Targeted axillary dissection (TAD) after Neoadjuvant chemotherapy (NACT) for Node-positive breast cancer: systematic review and pooled analysis. Cancers (Basel). 13(7):1539 33810544 10.3390/cancers13071539 PMC8037051

[CR36] Tolaney SM et al (2015) Adjuvant paclitaxel and trastuzumab for node-negative, HER2-positive breast cancer. N Engl J Med 372:134–141 25564897 10.1056/NEJMoa1406281 PMC4313867

[CR37] Tsai WC, Lin CK, Wei HK, Yu BL, Hung CF, Cheng SH, Chen CM (2013) Sonographic elastography improves the sensitivity and specificity of axilla sampling in breast cancer: a prospective study. Ultrasound Med Biol 39:941–949 23465139 10.1016/j.ultrasmedbio.2012.12.013

[CR38] Tutt ANJ et al (2021) Adjuvant olaparib for patients with BRCA1-or BRCA2-mutated breast cancer. N Engl J Med. 384(25):2394–2405 34081848 10.1056/NEJMoa2105215 PMC9126186

[CR39] Uematsu T, Sano M, Homma K (2001) In vitro high-resolution helical CT of small axillary lymph nodes in patients with breast cancer: correlation of CT and histology. AJR Am J Roentgenol 176:1069–1074 11264113 10.2214/ajr.176.4.1761069

[CR40] Valente SA, Levine GM, Silverstein MJ, Rayhanabad JA, Weng-Grumley JG, Ji L, Holmes DR, Sposto R, Sener SF (2012) Accuracy of predicting axillary lymph node positivity by physical examination, mammography, ultrasonography, and magnetic resonance imaging. Ann Surg Oncol 19:1825–1830 22227922 10.1245/s10434-011-2200-7

[CR41] van der Voort A, van Ramshorst MS, van Werkhoven ED, Mandjes IA, Kemper I, Vulink AJ et al (2021) Three-year follow-up of neoadjuvant chemotherapy with or without anthracyclines in the presence of dual ERBB2 blockade in patients with ERBB2-positive breast cancer: a secondary analysis of the TRAIN-2 randomized, phase 3 trial. JAMA Oncol 7(7):978–984 34014249 10.1001/jamaoncol.2021.1371 PMC8138752

[CR42] Veronesi U, Cascinelli N, Mariani L, Greco M, Saccozzi R, Luini A, Aguilar M, Marubini E (2002) Twenty-year follow-up of a randomized study comparing breast-conserving surgery with radical mastectomy for early breast cancer. N Engl J Med 347:1227–1232 12393819 10.1056/NEJMoa020989

[CR43] Veronesi U et al (2003) A randomized comparison of sentinel-node biopsy with routine axillary dissection in breast cancer. N Engl J Med 349:546–553 12904519 10.1056/NEJMoa012782

[CR44] von Minckwitz G et al (2019) Trastuzumab emtansine for residual invasive HER2-positive breast cancer. N Engl J Med 380:617–628 30516102 10.1056/NEJMoa1814017

[CR45] Wang L, Li Y, Li J, Wang T, Xie Y, He Y, Fan Z, Ouyang T (2021) Computed tomography reconstruction for evaluating response in axillary lymph nodes of breast cancer after neoadjuvant chemotherapy. Clin Transl Oncol 23:240–245 32519177 10.1007/s12094-020-02411-w

[CR46] Yuen S, Yamada K, Goto M, Sawai K, Nishimura T (2004) CT-based evaluation of axillary sentinel lymph node status in breast cancer: value of added contrast-enhanced study. Acta Radiol 45:730–737 15624516 10.1080/02841850410001088

[CR47] Zhao YX, Liu YR, Xie S, Jiang YZ, Shao ZM (2019) A nomogram predicting lymph node metastasis in T1 breast cancer based on the surveillance, epidemiology, and end results program. J Cancer 10:2443–2449 31258749 10.7150/jca.30386 PMC6584352

[CR48] Zhou P, Wei Y, Chen G, Guo L, Yan D, Wang Y (2018) Axillary lymph node metastasis detection by magnetic resonance imaging in patients with breast cancer: a meta-analysis. Thorac Cancer 9:989–996 29877048 10.1111/1759-7714.12774 PMC6068453

